# Hypoxia: The “Invisible Pusher” of Gut Microbiota

**DOI:** 10.3389/fmicb.2021.690600

**Published:** 2021-07-22

**Authors:** Ni Han, Zhiyuan Pan, Guangwei Liu, Ruifu Yang, Bi Yujing

**Affiliations:** ^1^State Key Laboratory of Pathogen and Biosecurity, Beijing Institute of Microbiology and Epidemiology, Beijing, China; ^2^Key Laboratory of Cell Proliferation and Regulation Biology, Ministry of Education, Institute of Cell Biology, College of Life Sciences, Beijing Normal University, Beijing, China

**Keywords:** hypoxia, gut microbiota, high altitude, tumor microenvironment, COVID-19

## Abstract

Oxygen is important to the human body. Cell survival and operations depend on oxygen. When the body becomes hypoxic, it affects the organs, tissues and cells and can cause irreversible damage. Hypoxia can occur under various conditions, including external environmental hypoxia and internal hypoxia. The gut microbiota plays different roles under hypoxic conditions, and its products and metabolites interact with susceptible tissues. This review was conducted to elucidate the complex relationship between hypoxia and the gut microbiota under different conditions. We describe the changes of intestinal microbiota under different hypoxic conditions: external environment and internal environment. For external environment, altitude was the mayor cause induced hypoxia. With the increase of altitude, hypoxia will become more serious, and meanwhile gut microbiota also changed obviously. Body internal environment also became hypoxia because of some diseases (such as cancer, neonatal necrotizing enterocolitis, even COVID-19). In addition to the disease itself, this hypoxia can also lead to changes of gut microbiota. The relationship between hypoxia and the gut microbiota are discussed under these conditions.

## Introduction

For human being or animal, there are two kinds of environment: internal environment and external environment (Gao, 2019). The environment where cells directly live in the body is called internal environment, which is the place where cells directly metabolize and live. Oxygen and various nutrients needed for cell metabolism can only be absorbed from internal environment (Turner, 2009). Therefore, the internal environment is very important for the survival of cells and the maintenance of their normal physiological functions. The environment outside the human body, such as air, water, soil, food, geology and landform, is collectively referred to as the external environment (Castro Rocha et al., 2020). Hypoxia in the body, happen not only from external conditions (mayor from high altitude) which caused systemic hypoxia, but also from internal environment (such as colon cancer, some lung diseases etc.) causing body local hypoxia (MacIntyre, 2014; Taylor and Colgan, 2017). Once the body is in a state of hypoxia, whether it is caused by external environment or internal diseases, it will cause reversible or irreversible body damage (Bogdanovski et al., 2017).

Hypoxia can lead to dizziness, headaches, tinnitus, weakness of limbs, nausea, vomiting and other symptoms. The symptoms of worsening hypoxia include gradual cessation of consciousness, purple skin all over the body, decreased blood pressure, dilated pupils, coma, and finally death due to breathing difficulties, cardiac arrest, and hypoxic asphyxiation. Altitude sickness can occur when the body undergoes a change in altitude (Zhang et al., 2020). Some diseases, such as colorectal and liver cancer, neonatal necrotizing enterocolitis (NEC), and the currently urgent COVID-19, involve varying degrees of anoxia (Zhou et al., 2020). Clinical treatment of hypoxia focuses on improving the anoxic condition and increasing the blood oxygen saturation. Under hypoxia, cells produce a transcriptional activator known as hypoxia-inducible factor-1 (HIF-1) (Lee et al., 2019). The α subunit of Hif-1 is the active subunit of Hif-1 and is a key factor in hypoxia signaling pathway. In general, expression of Hif-1α cannot be detected in cells with normal oxygen saturation. However, under hypoxia condition, Hif-1α subunit and Hif-1β subunit can form active heterodimer, which can be transferred to the nucleus to participate in the transcription of various genes, thereby regulating hypoxia state in the body. The expression of HIF can help keep the internal environment of cells and tissues stable under hypoxia condition, so as to adapt to hypoxia state (Tirpe et al., 2019).

Microenvironment is mainly reflected as an internal environment of the human body. Microenvironment directly determines the active state of individual microorganisms, and the change of macroenvironment often leads to the rapid change of microenvironment and affects the active state of microbial community, which shows the phenomenon of “internal and external differences” to a certain extent (Bloom and Zaman, 2014; Albert-Bayo et al., 2019). The human intestinal microenvironment is relatively complex. The intestinal microbiota includes one hundred million microorganisms with more than 1,000 species, most of which are obligate anaerobes (Faith et al., 2013). Under normal conditions, the intestinal microbiota, mucosal barrier and epithelial barrier constitute a complete intestinal defense mechanism that can resist invasion of pathogenic bacteria by inhibiting the displacement of commensal bacteria and endotoxins in the intestinal tract, increasing mucus secretion, enhancing the intestinal mechanical barrier, and inhibiting pathogenic bacterial growth (Fukuda et al., 2011). The intestinal microbiota remains in a dynamic, balanced state and can play roles in metabolism, nutrition, barrier protection and maintenance of normal immune function (Olszak et al., 2012; Smith et al., 2013; Ramezani and Raj, 2014). When the human body undergoes, barrier is the first to be affected. Some conditional pathogens have the opportunity to grow quickly. These anaerobic bacteria (Han, 2015; O’Neill et al., 2015; Figueredo et al., 2018) destroy the intestinal microecological barrier, cause the intestinal microbiota disordered, and finally lead to systemic inflammation. Evidence shows that the intestinal microecology plays an important role when the human body becomes hypoxic. Intestinal microbial ecosystem can affect vascular physiology (Kiouptsi et al., 2019) and even determine the degree of myocardial infarction (Lam et al., 2012; Reinhardt et al., 2012). Here, we review the relationship between intestinal microbiota and hypoxia from two aspects: altitude-associated hypoxia (external environment), and disease-related hypoxia (internal environment).

## Altitude-Associated Hypoxia and the Gut Microbiota

### Acute Hypobaric Hypoxia

The high altitude, low air pressure, thin air, and low oxygen content in plateau areas cause changes in the digestive system upon entering the plateau, thus destroying the gastrointestinal barrier and causing dysfunction, and resulting in bacterial displacement and intestinal microbiota imbalance. Oxygen is the main factor affecting human behavior and activities under hypoxia conditions (Basnyat and Murdoch, 2003). Studies have shown that the intestine is the central organ involved in the body’s stress response (Wilmore et al., 1988). Altitude stress can significantly reduce the occurrence of small intestinal migratory complex movement during the inter-digestive period (Yoshimoto et al., 2004). The reduced intestinal peristalsis affects the ability of bacteria to be flushed downward, making it easier for them to colonize the small intestine (Jo et al., 1987).

Humans and their gut microbiota maintain a dynamic coexistence and a mutually beneficial relationship, but this relationship will change when exposed to environmental pressure. This can destroy the integrity of the intestinal mucosal barrier, resulting in intestinal damage and bacterial antigen translocation from the intestinal cavity to the circulatory system, and leading to systemic inflammation and increasing susceptibility to diseases (Turner, 2009; van Wijck et al., 2012). As a physiological source of stress, hypoxia tends to occur at altitudes of >2500 m. The characteristics of hypoxia in plateau environments affect and restrict the activities and behaviors of organisms, thus changing the intestinal microorganismal composition.

**FIGURE 1:**
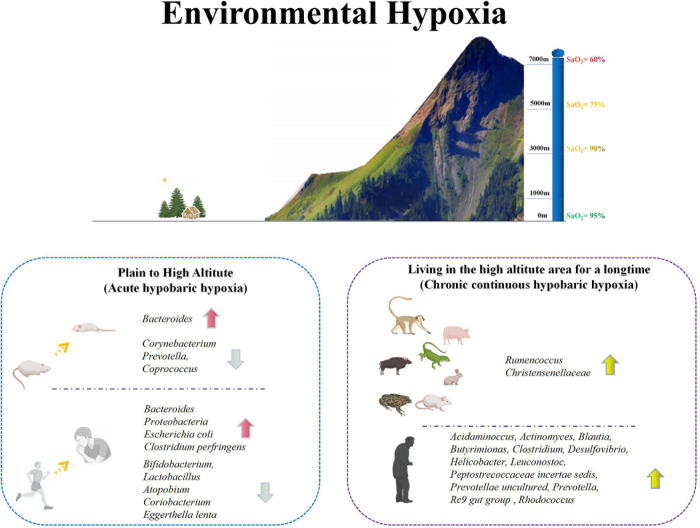
Changes of intestinal microbiota in animals and humans under environmental hypoxia. Environmental hypoxia can be divided into acute hypobaric hypoxia and chronic hypobaric hypoxia. Acute hypobaric hypoxia usually refers to the environment from plain to plateau. In this case, the intestinal microorganisms in animals and human will change greatly. In animals, *Bacteroides* increased significantly, while *Corynebacterium*, *Prevotella* and *Coprococcus* decreased significantly. The acute changes in human were mainly reflected in the significant increase of *Bacteroides*, *Proteobacteria, Escherichia coli* and *Clostridium perfringens*, and the significant decrease of some probiotics, including *Bifidobacterium, Lactobacillus, Atopodium, Coriobacterium*, and *Eggerthella lenta*. Chronic hypobaric hypoxia mainly refers to animals and people living in plateau environment for a long time. There is a high abundance of *Rumencoccus* and *Christensenellaceae* in animals living in plateau environment for a long time. However, there are some differences between human living in plateau environment and the plain, mainly in the higher abundance of *Acidaminocus, Actinomyces, Blautia, Butyrimionas, Clostridium, Desulfovibrio, Helicobacter, Leuconostoc, Peptostrecoccaceae incertae sedis, Prevotellae uncultured, Prevotella, Rc9* gut group and *Rhodococcus*. Based on the above two kinds of hypoxia conditions, it shows that hypoxia can indeed affect the intestinal microorganisms *in vivo*.

Research showed that mountaineering expeditions in the Himalayas by explorers exposed to high altitudes above 5000 m have increased the number of potentially pathogenic gram-negative bacteria from the gamma subbranch of *Proteobacteria*, particularly certain *Enterobacteriaceae*, such as *Escherichia coli*. The increase in this group of potential pathogens paralleled the decline of *Bifidobacterium*. The reduction of beneficial *Bifidobacteria* can lead to instability of intestinal microbiota (Gibson and Wang, 1994), which brings health risks to high-altitude mountaineers. This study suggests that the changes in intestinal microbiota caused by altitude exposure above 5000 m may be related to hypoxia stress (Kleessen et al., 2005). Simultaneously other studies (Adak et al., 2013) showed that the total number of anaerobic bacteria in the fecal microbiota of the high altitude group increased, while the total number of aerobic bacteria significantly decreased, and the protease and polyphenol hydrolase produced by different microbial communities were positive, while phosphatase produced negative. Enzymes such as amylase, protease, alkaline phosphatase and β-glucuronidase produced during microbial domestication at high altitudes are also elevated, and they demonstrated that hypoxic environments at high altitudes may alter the composition and activity of intestinal microbiota, leading to gastrointestinal dysfunction (Adak et al., 2013). Also people living at an altitude of 3505 m showed that the total oxygen demand of the population at this altitude was lower than that of people living on the plain, and some beneficial bacteria (*Bifidobacterium* and *Lactobacillus*) and some potential harmful bacteria (*Escherichia coli* and *Clostridium perfringens*) and anaerobic bacteria were increased in the body (Figure 1; Adak et al., 2013).

In addition to humans, animals entering hypoxia environment also showed the changes of bodies and gut microbiota. Skiers, religious pilgrims, hikers, mountain climbers, and military personnel have experienced altitude sickness and disease associated with a lack of oxygen at high altitude (Butterfield et al., 1992; Westerterp et al., 1992; Camp et al., 2009). The ratio of total aerobic to anaerobic bacteria in plain mice (healthy male albino rats, average body weight 145 ± 7 g) changed from 1:2.79 to 1:7.34 under exposure to a high-altitude environment for 30 days (Adak et al., 2014b). The total number of anaerobes increased from 6.02 to 8.04 log_10_ colony-forming units/g in the experimental group, which was 105 times higher than that of the control group (Adak et al., 2014a). Environmental pressure at high altitude leads to hypoxia, which reduces oxygen transport to surrounding tissues, leading to cellular hypoxia (Adak et al., 2013). Therefore, the microenvironment in the intestinal lumen is conducive to anaerobic growth.

Exposure to hypobaric hypoxia can increase gastrointestinal inflammation and permeability in rodents, accompanied by dynamic changes in intestinal microbial composition and activity (Zhou et al., 2011; Adak et al., 2014b; Xu et al., 2014; Zhang et al., 2015). When rats rapidly enter the plateau, the metabolic activity in their fecal suspensions is significantly reduced, and their intestinal microbiota composition is significantly altered. α diversity analysis showed that the Sobs, Chao, ACE and Shannon index values of rats that entered a high-altitude plateau were significantly lower than those of the control, while the Simpson index increased significantly, indicating that the numbers and species of intestinal microbiota were significantly reduced in the rats in a plateau environment. Compared with the control group, the abundance of *Bacteroides* increased significantly, and the number of *Prevotella* decreased in the plateau group (Zhang J. et al., 2018). During environmental hypoxia in mice, the *Bacteroidetes* abundance increased at both the family and genus levels from days 1 to 14 in mice with hypoxia inducible factor-1 (HIF-1) deficiency in their bone marrow cells. This difference was significant compared with that of the wild-type group (Han N. et al., 2020) and was consistent with previous findings that *Bacteroidetes* was more abundant in HIF-1-deficient mice than in wild-type mice (Sun et al., 2020).

Under normal conditions, the intestinal microbiota remains in a balanced state with the human body and the external environment. However, in plateau areas >3000 m above sea level, most people cannot immediately adapt to the low air pressure and low-oxygen environment, and digestive dysfunction in patients with altitude sickness is an important complication.

### Chronic Hypobaric Hypoxia

Scientists have studied animals and humans who have lived in high-altitude areas for 1000 years to determine how these high-altitude residents overcome the challenges of living in a low-oxygen environment. The intestinal microbiota of herbivorous Tibetan antelope includes *Klebsiella pneumoniae*, *Streptococcus suis*, *Streptococcus sanguineus*, *Streptococcus lutei*, *Streptococcus vasculitis*, and *Streptococcus infantis*. Additionally, 34% of intestinal microbiota species are considered potentially new species in known genera (Bai et al., 2018). The cecal bacterial community in *Ochotona curzoniae* mainly comprises *Scleroderma* and *Bacteroides* (Li et al., 2016b), which is consistent with that of Tibetan antelope.

Population data showed that the intestinal microbiota in Han people decreased sharply after several years of migration from low to high altitudes (Li and Zhao, 2015). After living for long times on a plateau, Tibetan people’s unique lifestyle has resulted in their having much lower incidence rates of adenoma, colon cancer and other intestinal diseases than those of people living in low-altitude areas. 16S rRNA high-throughput sequencing showed that compared with other ethnic groups, Tibetans had rich intestinal microbiota, including *Enterococcaceae*, *Prevotella*, and *Enterococci* (Li et al., 2016c). The number of microbial genes in Tibetans was 4.9 times higher than that of Han populations (Li et al., 2020). At the genus level, the relative abundances of 13 genera (*Acidaminoccus*, *Actinomyces*, *Blautia, Butyricimonas*, *Clostridium*, *Desulfovibrio*, *Helicobacter*, *Leuconostoc*, *Peptostrecoccaceae incertae sedis*, *Prevotella uncultured*, *Prevotella*, *Rc9* gut group, and *Rhodococcus*) from people living on plateaus were significantly higher than those of people living on plains (Li and Zhao, 2015). The bacterial levels of *Firmicutes*, *Clostridium* and *Ruminococcus* in the gut microbiotas of Tibetans living in high-altitude areas were higher than those of low-altitude Tibetans (Figure 1; Li et al., 2016c; Lan et al., 2017).

For plateau animals, researches compared the differences in intestinal microbiota between plateau pika and the low-altitude dauricus. *Scleroderma* was the most abundant genus in the plateau pika, and *Prevotella*, *Oscillospira*, *Ruminococcus*, and *yrc22* were abundant in the plateau pika. while *Proteobacteria*, *Actinobacteria*, and *Verrucomicrobia* were abundant in Ochotona dauricus. The Shannon index and evenness of the plateau pika were significantly higher than those of the dauricus, and eight genera, including *Streptococcus* and *Pseudomonas*, increased with the altitude. Therefore, altitude is important in forming the intestinal microbial community diversity in pika (Li et al., 2018, 2019). Interesting, *Ruminococcus* is a common group in the intestinal microbiota of all plateau herbivores in the same high-altitude areas, and results indicate that *Ruminococcus* is more abundant in the intestines of plateau pika than in low-altitude pika (Li et al., 2016a). *Ruminococcus* can produce short-chain fatty acids (SCFAs) (Hooda et al., 2012), whose levels can directly affect energy metabolism in the colonic epithelium (Canfora et al., 2015). SCFA levels in Yak rumens are higher than those in cattle, thus helping them adapt to high altitudes (Zhang et al., 2016). Other animals, such as black-necked cranes (*Grus nigricollis*) (Wang et al., 2020), lizards (*Phrynocephalus vlangalii*) (Zhang W. et al., 2018), Chinese Rhesus macaques (*Macaca mulatta*) (Zhao et al., 2018; Wu et al., 2020b), Bufo gargarizans (Xu et al., 2020), Tibetan chickens (Zhou et al., 2016) and Tibetan pigs (Zeng et al., 2020), also have high abundances of *Ruminococcus* in their guts (Table 1). These data indicate the importance of *Ruminococcus* in plateau adaptation and hypoxia-tolerant animals.

**TABLE 1:** Gut microbiomes of animals living in plateau environments.

**Animal model**	**Design**	**Microbiota method**	**Results-microbiota**	**References**
Tibetan antelope	*N* = 104; measured by collected	16S rRNA gene sequencing	*Klebsiella pneumoniae, Streptococcus suis, S. sanguinis, S. lutetiensis, S. anginosus, and S. infantis.*	Bai et al., 2018
Pika	Plateau pikas (*N* = 76) and *Daurian pikas* (*N* = 26); After animals were euthanized and dissected, stomach and cecal contents were immediately collected from the wild pikas and then frozen at once in –20°C portable refrigerator	16S rRNA gene sequencing	At genus level, *Prevotella, Oscillospira, Ruminococcus, YRC22, Lactobacillus, Coprococcus*, and *CF231* were enriched in Plateau pikas, *while Akkermansia, Streptococcus*, and *Pseudomonas* were more abundant in *Daurian pikas*.	Li et al., 2018
Black-necked cranes (*Grus nigricollis*)	*N* = 33; measured by collected	16S rRNA gene sequencing	At the phylum level, the black-necked cranes’ gut microbiota was dominated by *Firmicutes* (58.70%), *Proteobacteria* (37.09%), *Actinobacteria* (3.23%), and *Bacteroidetes* (0.36%).	Wang et al., 2020
Lizard *Phrynocephalus vlangalii*	*N* = 15; measured by collected	high-throughput sequencing	*Bacteroidetes* in the intestines of P. vlangalii lizards tended to be higher with altitude	Zhang W. et al., 2018
Bufo gargarizans	*N* = 88; measured by collected	high-throughput sequencing	*Bacteroidaceae, Lachnospiraceae, Erysipelotrichaceae, Coxiellaceae, Spirochaetaceae*, and *Mycoplasmataceae.*	Xu et al., 2020
Chinese Rhesus Macaques (*Macaca mulatta*)	*N* = 36; measured by collected	16S rRNA gene sequencing	At the phylum level, the whole rhesus macaque gut microbiota was largely dominated by *Firmicutes* (*x* = 53.60 ± 8.65%), *Bacteroidetes* (*x* = 33.60 ± 11.87%), and *Tenericutes* (*x* = 6.10 ± 4.91%); other represented phyla were *Cyanobacteria* (*x* = 2.40 ± 3.18%), *Actinobacteria* (*x* = 1.55 ± 1.29%), *Spirochaetae* (*x* = 1.36 ± 1.83%), and *Proteobacteria* (*x* = 0.75 ± 0.44%)	Wu et al., 2020b
Tibetan chickens	*N* = 15; measured by collected	16S rRNA gene sequencing	*Christensenellaceae* were relatively more abundant in the Tibetan Chickens, as were *Subdoligranulum, Spirochaeta*, and *Treponema.*	Zhou et al., 2016
Tibetan pigs	*N* = 102; measured by collected	16S rRNA gene sequencing	Unclassified *Enterobacteriaceae* and *Comamonas* spp. (only in high-altitude Tibetan pigs)	Zeng et al., 2020

These studies suggest that the plateau environment is associated with an altered intestinal microbiota in both animals and humans. Hypoxia is an important characteristic of plateau environments [although there may be confounding factors, “including food (nutrition), temperature, air pressure, etc.]. However, studies on population data are limited, and no reports exist regarding which intestinal microbiota can alleviate the hypoxic effects. Researchers must determine the important bacterial microbiota via randomized controlled trials to clarify the roles of intestinal microorganisms.

## Internal Hypoxia and Gut Microbiota

### Tumor-Related Hypoxia

The tumor microenvironment comprises tumor cells, resident and recruited host cells (cancer-related stromal and immune cells), and secreted products of corresponding cells (e.g., cytokines and chemokines) and the extracellular matrix. It also comprises non-cellular components and may include tumor-microenvironment metabolites and specific environments (Germa Lluch et al., 1991). Blood vessels are prioritized during tumoral growth. Countless blood vessels and capillaries exist in the human body. Most tumor cells spread in the bone marrow and depend on capillaries for growth. When microvessels begin growing, quiescent tumor cells can be stimulated to develop into aggressive tumors. As tumors continuously grow, their malignant transformation becomes a vicious cycle that constantly disrupts the body’s homeostasis. Tumor cells can continuously build new nutritional metabolism networks by inducing angiogenesis to promote tumor cell growth (Folkman, 2003), which is known as the “seed and soil” hypothesis (Potter and Wisniewski, 2012). When the original blood vessels can no longer fully perfuse the tumor, oxygen levels drop, and an environment of hypoxia and malnutrition forms where metabolic byproducts accumulate and immunosuppression is regulated. Hypoxia is a common sign of malignant tumor growth (Chae et al., 2016). Hypoxia initiates HIF to induce blood vessel proliferation, thus making the environment more hypoxic, and the tumor microenvironment can alter the intestinal microbiota.

#### Colorectal Cancer

Intestinal microenvironment changes may be a cause of colorectal cancer (Jawad et al., 2011; Zoratto et al., 2014). An important feature of intestinal microbiota imbalance in patients with colorectal cancer is an increase in conditional pathogens (Szabo et al., 2010; Castillo-Carranza et al., 2013). Intestinal imbalance can lead to mucus production in the intestinal mucosa, abnormal intestinal mucosal epithelial hyperplasia, and increased intestinal permeability, this affects the expression of related antibacterial proteins, largely destroying the barrier function of the intestinal mucosa in patients’ bodies. Studies have shown that the distribution characteristics of the intestinal microbiotas of colorectal cancer patients differ greatly from those of healthy people. *Firmicutes* and *Fusobacteria* are overexpressed in the intestines of colorectal cancer patients, while *Proteobacteria* is reduced. In addition, compared with tissues adjacent to the cancer tissue, *Lactococcus* and *Fusobacteria* show higher abundances, while *Pseudomonas* and *Escherichia-Shigella* are reduced (Gao et al., 2015). The abundances of *Clostridium tetani*, *Clostridium sphaeroides*, and pathogenic *E. coli* are significantly higher in the intestines of patients with colorectal cancer than in those of healthy controls, and the numbers of butyrate-producing bacteria are significantly reduced (Szabo et al., 2010; Huang and Mucke, 2012). *Fusobacterium nucleatum* is a prominent conditional pathogen in the occurrence and development of colorectal cancer (Castellarin et al., 2012). These increases in pathogenic bacteria and decreases in butyrate-producing bacteria significantly increase the risk of morbidity in patients with colorectal cancer (Potter and Wisniewski, 2012). The results showed that NaB (Sodium butyrate) not only inhibited the liver metastasis rate of tumor cells, but also improved the dysbacteriosis of CLM mice, including reducing the abundance of pathogenic bacteria, increasing the abundance of beneficial bacteria such as short chain fatty acid producing bacteria, and reducing the *Firmicutes*/*Bacteroides* ratio. At the same time, NaB increased NKT cells and Th17 cells, decreased Treg, and improved the anti-tumor immune response in the liver of CLM mice (Ma et al., 2020). Therefore, NaB can reduce toxic bacterial products and increase beneficial bacterial metabolites, highlighting the potential of NaB in the treatment of CLM.

#### Liver Cancer

The intestines are closely related to the liver, leading to the “intestine-liver axis” theory (Arab et al., 2018). The mesenteric vein is connected to the portal vein, which takes up approximately 70% of the blood supply. Nutrients and metabolic waste absorbed by the intestines enter the liver through the portal vein (Compare et al., 2012; Dapito et al., 2012; Yoshimoto et al., 2013). When the small intestines absorb too many lipids, excess lipids accumulate, which can cause stem cell steatosis in the liver, leading to non-alcoholic fatty liver disease (Chu et al., 2019). Intestinal microbiota and their metabolites can also affect liver pathology (Lambert et al., 2015). Studies have shown that when non-alcoholic fatty liver gradually develops into non-alcoholic steatohepatitis (NASH), harmful microbiota in the intestines grow excessively, leading to a disorder of the intestinal microecology (Flass et al., 2015).

Changes in the intestinal microbiota of patients with non-alcoholic hepatitis may lead to the production of reactive oxygen species, which can lead to NASH-DNA damage and increase the risk of liver cancer. Hypoxia microenvironment is a common feature of solid tumors, and hepatoma cells have strong oxygen tolerance. In hypoxia environment, hepatoma cells activate target genes containing a hypoxia response element (HRE) through HIF-1 transcription (in human tumor cells, these gene products are greatly increased) to cause a series of tumor cells to respond to hypoxia (Kaneoka et al., 2003). HIF-1 can also enhance the ability of glycolysis to make tumor adapt to hypoxia environment quickly (Kakaty et al., 2015). Tumor cells have stronger survival ability than normal tissue cells in hypoxia environment, thus enhancing the ability of tumor invasion and metastasis. The growth of tumor cells and hypoxia in tumor microenvironment form a positive feedback effect, which increases the degree of hypoxia in the body. Simultaneously, the number and structure of intestinal microbiota in patients with pre-liver cancer (i.e., non-alcoholic hepatitis) changed, and the prevalence of intestinal bacterial overgrowth was significantly higher than that in healthy people (Shanab et al., 2011).

In patients with non-alcoholic hepatitis, the proportion of *Bacteroides* was lower, anaerobic bacteria such as *Lactobacillus* and *Bifidobacterium* were decreased, and the intestinal aerobic bacteria, such as *Coccus* and *Enterobacteria*, were increased compared with those of patients with simple fatty liver and of healthy controls (Mouzaki et al., 2013). When non-alcoholic hepatitis occurs, intestinal permeability and intestinal bacteria increase. During this time, the liver is the body’s first barrier against exposure to toxins and antigens. Gram-negative bacteria are the primary intestinal endotoxins. Under normal conditions, intestinal endotoxins pass through the liver to be detoxified, whereas the endotoxins in non-alcoholic hepatitis patients cannot be detoxified after entering the liver through the portal vein and thus enter the systemic circulation and damage the liver cells. Patients with non-alcoholic hepatitis will have intestinal microbiota imbalance and overgrowth of gram-negative bacteria, indicating that the imbalanced intestinal microecology may be involved in the development of non-alcoholic hepatitis (Neuschwander-Tetri, 2017). An imbalanced intestinal microecology can activate the lipopolysaccharide/Toll-like receptor-4 (LPS-TLR4) signaling pathway, increase the intestinal wall permeability, cause mesenteric epithelial cells to develop ischemic necrosis, and induce liver fibrosis (Hartmann et al., 2012). The composition of the intestinal microbiota of these patients is different from that of healthy people. Increased permeability of intestinal wall (Levy et al., 2017; Sepehri et al., 2017; Wree et al., 2018) is one of the main mechanisms by which the intestinal microbiota participates in the pathogenesis and progression of NAFLD.

### Hypoxia Induced by Neonatal Necrotizing Enterocolitis

Neonatal NEC is an acquired disease that is the most common and destructive gastrointestinal disease of premature infants (Ou et al., 2020), with NEC incidence of about 7% (Hackam and Caplan, 2018) and mortality of 10-30% (Alganabi et al., 2019). Hypoxia is one of the main causes of NEC, and the other causes include the immature intestinal function, and intestinal immune barrier damage of neonates. Classic NEC is a complex, multifactorial disease (Neu, 1996), some diseases such as congenital heart disease (Giannone et al., 2008), anemia (Patel et al., 2016), preeclampsia in pregnant women (Perger et al., 2016), and changes in intestinal microcirculation (Downard et al., 2011) can promote the occurrence of NEC, which are all related to hypoxia. NEC mainly induces intestinal necrosis from the mucosal layer and gradually involves the entire thickness of the intestinal wall, resulting in perforation. Neonatal respiratory distress, hypoxia and other conditions will make the intestinal wall vasoconstriction, resulting in intestinal mucosa hypoxia ischemia, and then restore oxygen supply, vasodilation and congestion, expansion of reperfusion will increase tissue damage and lead to NEC.

Some *in vitro* experiments have confirmed that the immature intestinal cells in infants are more prone to inflammation due to pathogenic stimulation (Nanthakumar et al., 2000; Claud et al., 2004). Intestinal microorganisms greatly influence the development of the gastrointestinal tract and maintenance of the mucosal surface integrity (Caicedo et al., 2005), but the intestinal barrier function in the immature intestines of premature infants is imperfect. Bacteria and their metabolites that were originally confined to the intestinal lumen can be displaced and spread to nearby organs and tissues (Uauy et al., 1991; Hintz et al., 2005), triggering a chain inflammatory response, leading to further intestinal epithelial damage (Viscardi et al., 1997; Halpern et al., 2002; Harris et al., 2005; Claud and Walker, 2008). High-throughput sequencing have found that the occurrence of NEC is related to increases in *Enterobacter*, *Fusobacterium*, *Shigella*, *Enterobacter sakazakii*, γ*-Proteobacteria* (Morrow et al., 2013; Zhou et al., 2015; Warner et al., 2016), and propionic acid. A reduction or lack of *Bacteroides*, *Clostridium*, and *Negativicutes* is also related (Morrow et al., 2013; McMurtry et al., 2015; Warner et al., 2016). Simultaneously *Clostridium* is an important member of the microbial community that can effectively prevent the expansion of symbiotic *Escherichia coli* (Rivera-Chavez et al., 2016), the depletion of *Clostridium* drives the expansion of pathogens.

One to two weeks before NEC was diagnosed, the proportions of *Proteobacteria* and *Actinobacteria* were significantly increased in NEC patients compared with those of the control group (Handl et al., 2011). High-throughput sequencing results revealed that *Proteobacteria* was increased by 32%, and *Firmicutes* was decreased by 32%, suggesting that the gut microbial community structure changed gradually before the onset of NEC (Simon and Daniel, 2011). The diversity index of the fecal microbiota in children with NEC was significantly lower than that of children without NEC (Wang et al., 2009; Morrow et al., 2013; Torrazza et al., 2013; McMurtry et al., 2015). This suggests that decreased abundances in the intestinal microbial diversity may be related to NEC (McMurtry et al., 2015).

Necrotizing enterocolitis mainly affects premature infants. The incidence is lower in full-term infants, accounting for < 10% of children with NEC. Therefore, establishing intestinal microbiota in infants and young children is important to their health and enables diagnosing and treating diseases early by changing and regulating the intestinal microbiota.

### Hypoxia Induced by COVID-19

Corona Virus Disease 2019 is an acute infectious pneumonia. When the novel *Coronavirus* invades the human body, the organs most seriously affected are the lungs. In most lung diseases, blood oxygen saturation decreases as the lung disease is aggravated. Other changes include lung stiffness, fluid filling, and rising carbon dioxide levels because the lungs cannot effectively flush out the carbon dioxide. In severe cases of COVID-19, people have difficulty breathing, their lungs are damaged, and blood oxygen saturation drops to between 70 and 80% and below 50% in some cases. At this point, the lungs are severely damaged, and the lack of oxygen can damage other organs, such as the heart, kidneys, intestines and brain, then rapidly develop into respiratory failure. The blood oxygen saturation in the body is decreased, the hypoxic phenotype is obvious, and the patients with new coronary disease are suffering from respiratory distress. CT shows multiple lung diseases. Compared with SARS in 2003, patients with new coronary pneumonia progressed faster in respiratory failure, and the development of hypoxia was more obvious. Therefore, oxygen therapy is very important in the treatment of new coronary pneumonia. If the patient can ensure adequate oxygen supply in the early stage, the chance of a sudden illness becoming severe will be less. Simultaneously, the intestinal microbiota is associated with respiratory tract microbial infections and can influence the occurrence and development of diseases through the intestinal-lung axis (Marsland et al., 2015).

Imbalances in the intestinal microbiota reduce the immune defenses in the respiratory tract, making it easier for respiratory viruses to invade the body (Ichinohe et al., 2011). And patients with intestinal diseases are considered to be at high risk of SARS-CoV-2 infection (Iacucci et al., 2020; Vodnar et al., 2020). In addition, the pandemic data showed that SARS-CoV-2 RNA was found in stool samples of patients with COVID-19, including stool samples of several patients with negative upper respiratory tract test results (D’Amico et al., 2020; Han C. et al., 2020; Hindson, 2020; Pan et al., 2020; Wu et al., 2020a; Xiao et al., 2020). The diversity and abundances of the intestinal microbiota were significantly reduced in patients with COVID-19 and H1N1, compared with those of healthy people (Groves et al., 2018; Yildiz et al., 2018). The relative abundances of opportunistic pathogens in COVID-19 patients were increased and mainly included *Streptococcus*, *Rothia*, *Veillonella*, *Erysipelatoclostridium*, and *Actinomyces*. Changes in the intestinal mucosa and immune factors induced by *Actinomycetes* may aggravate the damage from intestinal diseases (Lin et al., 2017; Nahum et al., 2017). Clinical data showed that compared with the control group, COVID-19 patients without antibiotics were rich in opportunistic pathogens that can cause bacteremia (Zuo et al., 2020), including *Clostridium hathewayi*, *Actinomyces viscosus*, and *Bacteroides NorDII*. The study also showed that *Firmicutes* was correlated with COVID-19 severity and that *Firmicutes*, *Coprobacillus*, *Clostridium ramosum* and *C. hathewayi* were the top bacteria positively correlated with the severity of COVID-19. Further, *C. ramosum* and *C. hathewayi* are both highly correlated with human infectious diseases and bacteremia (Elsayed and Zhang, 2004; Forrester and Spain, 2014). The intestinal microbial ecosystems of critically ill COVID-19 patients are disordered and prone to secondary infections. Studies have shown that respiratory viral infections may be related to changes in the intestinal microbiota, thus making patients prone to secondary bacterial infections (Hanada et al., 2018; Yildiz et al., 2018). Therefore, these patients often die from secondary bacterial infections rather than from the viral infection. In treating COVID-19 patients, the intestinal microecological balance should be considered, and the intestinal bacteria should be adjusted to maintain homeostasis.

### Hypoxia Caused by Other Diseases

In addition to the above diseases, which are typical under hypoxic conditions, other common chronic diseases also induced body hypoxia. Chronic obstructive pulmonary disease (COPD) is a respiratory disease characterized by chronic airway inflammation. The pulmonary characteristics of COPD primarily include reversible airflow limitation and progressive development. When airflow limitations lead to insufficient inhaled gases and disordered ventilation and blood flow, patients become deprived of oxygen. Among them, smoking is an important risk factor of COPD. The prevalence of COPD in smokers is 2-8 times higher than that in non-smokers (Tashkin, 2015; Peiffer et al., 2018; Duffy and Criner, 2019). Patients with COPD also have chronic dyspnea, coughing, sputum, and chest tightness, and their bodies will be in a hypoxic state for a long time, resulting in pathological changes. Studies have found that abnormal bacterial microbiota in the lungs can migrate to the intestinal tract through the blood circulation and destroy the relative balance of the intestinal microbiota (Chung, 2017; He et al., 2017). In clinical cases, patients with lung diseases commonly exhibit an intestinal microbiota imbalance. One study found that 40.26% of COPD patients had an intestinal microbiota imbalance (Huang et al., 2017) and that the number of intestinal probiotics in COPD patients was significantly lower than that in healthy people. Additionally, the frequency of acute COPD attacks is more closely related to decreases in *Bifidobacteria* and *Lactobacillus*.

Bronchial asthma is a common chronic lung disease with hypoxia and is a heterogeneous disease characterized by chronic airway inflammation involving various cells and cellular components. This chronic inflammation is associated with airway hyperresponsiveness, which causes symptoms such as hypoxia and dyspnea when the airway hyperresponsiveness and airway contraction are severe. Asthma is a common multiple respiratory diseases (Vora, 2014), repeated attacks will lead to insufficient blood supply to the brain. After the attack of asthma, because of bronchospasm will lead to hypoxia, lung gas of patients can’t pass normally (Boulet and Boulay, 2011). And severe persistent state of asthma itself can cause arrhythmia and shock due to the influence of hypoxia (Mims, 2015). Common respiratory diseases are associated with intestinal diseases, and use of antibiotics to alter the intestinal microbiota can make patients more susceptible to allergic asthma (Russell et al., 2012). An on-the-spot study found that the intestinal microbiota in asthmatic patients was significantly altered at the molecular level, the diversity was significantly reduced, and the structural changes in the intestinal microbiota were associated with asthma (Vael et al., 2008). *Bifidobacterium* is an intestinal probiotic that fights harmful bacteria in the human body (Santosa et al., 2006). Animal experiments have shown that administering *Bifidobacteria* combined with peptic oligosaccharides can inhibit lung airway inflammation, reduce T-cell activation, regulate pattern recognition receptors and cytokine expression and reduce airway remodeling (Sagar et al., 2014). Therefore, when severe lesions occur in patients’ lungs, timely attention should be paid to the patients’ intestinal microbiota to prevent the toxins secreted by the microbiota from entering the blood and to reduce lung damage.

Another common chronic hypoxic disease is obstructive sleep apnea hypopnea syndrome (OSAHS). OSAHS is a sleep respiratory disease of unknown etiology, characterized by chronic intermittent hypoxia (CIH) and subsequent reoxygenation. Because OSAHS patients have recurrent nocturnal hypoxia and hypercapnia at night due to apnea, OSAHS is a potentially fatal sleep respiratory disease and has become an important public health problem (Peppard et al., 2013). CIH plays a major role in the physiological damage of OSAHS patients (Lesske et al., 1997). CIH is a systemic injury that causes adverse reactions in various tissues and alters the intestinal microbiota (Moreno-Indias et al., 2015; Tripathi et al., 2018). Repeated hypoxia may be conducive to anaerobic bacterial proliferation. Even when normal oxygen is restored, the intestinal microbiota composition due to CIH remains affected. Studies have shown that CIH exposure can lead to delayed persistent low-grade endotoxemia in mice, and the abundance of LPS in the plasma is positively correlated with that of *Desulfovibrio* (Moreno-Indias et al., 2016). Bacterium-derived metabolites (e.g., SCFAs) are also affected by CIH exposure (Adnan et al., 2017; Li et al., 2017). Animal experiments have shown that the relative abundances of microorganisms were decreased significantly in guinea pigs exposed to CIH for 12 days and that the microbial community composition had changed, with a decreased relative abundance of *Firmicutes* and an increased relative abundance of *Bacteroides* (Lucking et al., 2018).

The overall microbial community structure of the experimental group was significantly changed after 6 weeks of intermittent hypoxia compared with the control group, and the α diversity of the intestinal microbiota was increased. The changes in the intestinal microbiota mainly included increased *Firmicutes* and fewer *Bacteroides* and *Proteobacteria* (Moreno-Indias et al., 2015). These results were consistent with those of previous studies. Another study showed that in rats exposed to intermittent hypoxia, the numbers of *Bacteroides* were decreased significantly, the numbers of *Firmicutes* and *Deferribacter* increased significantly, and the numbers of *Lactobacillus* and *Ruminococcus* were significantly negatively correlated (Moreno-Indias et al., 2016).

## Conclusion and Discussion

Oxygen has existed on earth for 250 million years, and humans have fully adapted to its existence. For humans, oxygen is the most important element in maintaining life. Japan’s highest medical authority, Hideki Noguchi, stated that “hypoxia is the source of all diseases.” Hypoxia can affect human health (Pilli et al., 2018). Different parts of the human body have different sensitivities and responses to hypoxia. The intestinal microbiota under a hypoxic environment is becoming an increasing concern. Changes in intestinal microbiota differ under different hypoxic conditions. In most cases, the emergence of a hypoxic environment will directly or indirectly affect the composition and abundance of the intestinal microbiota. Hypoxia will lead to the enrichment of *Prevotella in vivo*, and the corresponding low ratio of Bacteroides: *Prevotella* (Karl et al., 2018). Recent studies showed *Prevotella* abundance increases during oxidative stress, which promotes intestinal mucus barrier dysfunction and inflammation (Elinav et al., 2011; Scher et al., 2013).

Although increasing research is being conducted on hypoxia, the related gut microbiota under different hypoxic environments require further study. Clinicians must understand the complex relationship between hypoxia and intestinal microbiota and determine whether a further relationship exists between hypoxic mechanisms and intestinal microbiota. This information will provide effective and considerable insight for potential disease treatment combined with existing disease treatments. Although the intestinal microbiota has been extensively studied in the context of colorectal cancer and related cancers, few studies exist on its relationship with lung-related diseases and hypoxia, and this requires further exploration.

Many unsolved problems remain in the growing field of intestinal microbiota such as the impact of localized hypoxia on the intestinal tract and whether it affects the oxygen content of the intestinal environment to create a more favorable environment for intestinal anaerobic bacteria. This could result in the proliferation of intestinal anaerobic bacteria and a disordered intestinal microbiota. Other problems include whether intestinal bacteria that could serve as biomarkers exist in the intestinal tract and the mechanisms of hypoxia and the intestinal microbiota, including the hypoxia-related HIF pathway. In general, whether *in vivo* or *in vitro*, more studies are needed regarding cases of the body under hypoxia. The intestinal microbiota must be studied under hypoxic conditions to explore the mechanisms by which the intestinal microbiota handles hypoxia and to find new strategies for different diseases and to cope with high-altitude environments. Such strategies should include both targeted and individualized treatment for handling high-altitude hypoxia and treating different diseases involving hypoxia.

## Author Contributions

NH investigated the literature and wrote the manuscript. ZP investigated the literature. GL and RY contributed to revise the manuscript. BY provided overall directions and contributed to revising the manuscript. All authors contributed to the article and approved the submitted version.

## Conflict of Interest

The authors declare that the research was conducted in the absence of any commercial or financial relationships that could be construed as a potential conflict of interest.

## Abbreviations

HIF-1: hypoxia inducible factor-1
COVID-19: Corona Virus Disease 2019
SCFA: short-chain fatty acid
LPS: lipopolysaccharide
TLR4: Toll-like receptor-4
VEGF: Vascular Endothelial Growth Factor
CCHD: Cyanotic Congenital Heart Disease
NEC: necrotizing enterocolitis
COPD: chronic obstructive pulmonary disease
OSAHS: obstructive sleep apnea hypopnea syndrome
CIH: chronic intermittent hypoxia.
